# Larvicidal Activity of *Centaurea bruguierana* ssp. *belangerana* Against *Anopheles stephensi* Larvae

**Published:** 2011

**Authors:** Mahnaz Khanavi, Afsaneh Rajabi, Masoud Behzad, Abbas Hadjiakhoondi, Hassan Vatandoost, Mohammad Reza Abaee

**Affiliations:** a *Department of Pharmacognosy and Medicinal Plants Research Center, Faculty of Pharmacy, Tehran University of Medical Sciences, Tehran, 14155-6451, Iran.*; b *Department of Medical Entomology, School of Public Health and Institute of Health Research, Tehran University of Medical Sciences, Tehran, Iran.*

**Keywords:** *Centaurea bruguierana *ssp. *Belangerana*, Asteraceae, Larvicidal activity, *Anopheles stephensi *larvae

## Abstract

In this study, the total 80% of MeOH extract and also petroleum ether, CHCl_3_, EtOAc, *n*-BuOH, and the remaining MeOH fractions obtained by solvent-solvent fractionation of the whole flowering samples of *Centaurea bruguierana *(DC.) Hand.-Mzt. ssp. *belangerana *(DC.) Bornm. (Asteraceae), namely “Baad-Avard”, collected from Borazjan in Bushehr Province (Bushehr, Iran) were investigated for larvicidal activity against malaria vector, *Anopheles stephensi *Liston, according to WHO methods. The mortality rate of total extract and petroleum ether fraction in concentration of 40 ppm were 28% and 86% respectively and the other fractions were inactive. The probit regression analysis for the dose-response to petroleum ether fraction treatment of larvae exhibited the LC_50 _and LC_90_ values of 15.7 ppm and 48.3 ppm, respectively. As results showed, the larvicidal activity of the petroleum ether fraction would be due to the nonpolar compounds in the plant which further isolation and purification would obtain the more active compounds in lower concentrations useful for preparation of biological insecticides.

## Introduction

Malaria is the most important problem of developing countries and is still an endemic disease in more than 100 countries (1). According to the latest report of World Health Organization, it kills between 1.5-2.7 million people every year (2). Malaria is endemic in the south of Iran (3) and has always been considered as the most important vector-borne disease in Iran due to its socioeconomic effects on the population (4). Since the discovery of the insecticide dichlorodiphenyltrichloroethane (DDT) before the Second World War, the wide-spread use of synthetic insecticides for the control of pests as well as human disease vectors has led to concerns about their toxicity and environmental impact (5). Because of this, the search for new environmentally safe, target-specific insecticides based on natural plant products, is active throughout the world.

The genus *Centaurea *L. (Asteraceae, tribe Cardueae, subtribe Centaureinae) comprises ca. 600 species distributed widely from Asia, Europe and Tropical Africa to North America as aggressively invading weeds (6). This genus consists of 88 species in the Flora Iranica (7). *C. bruguierana *(DC.) Hand.-Mzt. ssp. *belangerana *(DC.) Bornm. (Sect. Tetramorphaea) – a 5-50 cm annual herb with purple spiny flowers-is distributed in Iran, Transcaucasia, Afghanistan, Pakistan, and Central Asia (7).

Many species of the genus *Centaurea *have long been used in traditional medicines to cure various ailments, *e.g. *diabetes, diarrhea, rheumatism, malaria, and also against coughs, as liver-strengthening, itch-eliminating and ophthalmic remedies (8-10). Various biological activities have been reported for *Centaurea *spp. so far including antiviral and antimicrobial for *C. solstitialis *ssp. *solstitialis *(11), antibacterial for *C. diffusa *(12), antifungal for *C. thessala *ssp. *drakiensis *and *C. attica *(13), antiplasmodial for *C. hierapolitana *(14), *C. eryngioides *(15) and *C. musimomum *(16), cytotoxic for *C. schischkinii *(17), *C. montana *(8) and *C. musimomum *(16), anti-inflammatory, analgesic and antipyretic for *C. ainetensis *(18), *C. chiliensis *(19), *C. tchihatcheffii *(20), *C. cyanus *(21) and *C. solstitialis *ssp. *solstitialis *(22), anti-peptic ulcer and anti-*Helicobacter pylori *for *C. solstitialis *ssp. *solstitialis *(23-25). In addition, a variety of secondary metabolites have been reported from different species of this genus including sesquiterpene lactones (11, 26-28), flavonoids (8, 10, 17, 29, 30), lignans (8, 17, 26) and alkaloids (8, 17).

As a part of our ongoing larvicidal screening of native Iranian plants, in this paper, we describe for the first time the larvicidal activity of *C. bruguierana *ssp. *belangerana *against *Anopheles stephensi *Liston which is the main malaria vector in southern Iran and resistant to DDT, dieldrin and malathion in this area (31-32).

## Experimental

*Plant material*

The whole flowering samples of *C. bruguierana *ssp. *belangerana, *namely “Baad-Avard”, were collected by “Agricultural Research and Natural Resources Center of Bushehr Province” from Borazjan (Borazjan, Bushehr Province) located in south of Iran, at an elevation of 70 m in June 2007 and identified by Professor Gh. Amin, Herbarium of the Faculty of Pharmacy, Tehran University of Medical Sciences (Tehran, Iran) where voucher specimen is deposited (6683-TEH).

*Extraction and solvent-solvent fractionation*

Dried whole flowering samples (300 g) were extracted with 80% methanol (MeOH, 6 × 1.5 l) in a percolator at room temperature for 2 weeks. The combined extract was concentrated to dryness under reduced pressure at 40°C. The MeOH extract was successively dissolved in 100 mL MeOH : H_2_O (7 : 3) and extracted with petroleum ether (4 × 200 mL), chloroform (CHCl_3_, 4 × 200 mL), H_2_O-saturated ethyl acetate (EtOAc, 4 × 200 mL) and H_2_O-saturated *n*-butanol (*n*-BuOH, 4 × 200 mL) in separatory funnel. Each fraction together with the remaining MeOH part after the solvent fractionation, were then evaporated to dryness under reduced pressure at 40°C for the purpose of test fraction. All solvents were purchased from Merck (Merck, Darmstadt, Germany).

*Mosquitoes*

*Anopheles stephensi *larvae used in this study were obtained from the laboratory of the “School of Public Health and Institute of Health Research” (Tehran University of Medical Sciences, Tehran, Iran) (originally from the malarious areas of Iran, Kazeroon, Fars province). They were reared under insectary conditions at 25 ± 1, 12/12 h (light**/**dark) photo-period and 50-70% relative humidity and were fed with 10% sucrose solution. The late 3^rd ^and early 4^th^ instar larvae were used for the tests. The sucrose solution was withdrawn from the cage, 14 h prior to the tests.

*Larvicidal assay*

The larvicidal activity of the total extract and fractions were assayed according to WHO methods (20). Preliminary testing was carried out to establish suitable stock solutions of the total extract and fractions as test samples. For each concentration, 4 replicates of 25 larvae were used. Each test run consisted of 224 mL water, 1 mL of test sample stock solution and 25 larvae in 25 mL water; so that the final volume was 250 mL. Finally, the resulted concentrations for test samples were as follows: 40, 20, 10, 5 and 2.5 ppm. In control runs, 1 mL of MeOH was added instead of test sample. Mortality was determined after a 24 h exposure period. In the analysis, both dead and moribund larvae were considered as dead. From the regression line between logarithmic dose and probit mortality, the LC_50_ was determined.

*Statistical analysis*

The percentage of mortality in the treated larvae was corrected relative to the control using Abbott’s formula (34). The mortality data were subjected to probit regression analysis according to Finney (35). The goodness of fit of the points to a straight line was tested by chi-square analysis. Data were computer analyzed through the probit plane procedure using *MicroProbit *software (version 3.0). From the regression line between the logarithmic dose and probit mortality, all the parameters including LC_50_, LC_90_, confidence interval (CI) and slope values were determined. Significant differences were determined through comparing the LC_50_ and 95% CI. The heterogeneity of the population was determined through the chi-square test. The regression line was plotted using Microsoft *Excel*.

## Results and Discussion

The extraction of plant powder and the fractionation of extract yielded 32.0 g of the total extract, 0.976 g petroleum ether fraction, 4.268 g CHCl_3_ fraction, 3.394 g EtOAc fraction, 3.485 g *n*-BuOH fraction and 13.077 g of the remaining MeOH fraction.

The results of the bioassay tests of the total methanolic extract and fractions on the *Anopheles stephensi *larvae are presented in Table 1.

**Table 1:** Larvicidal activity of total methanolic extract and fractions (conc. 40 ppm) of *C*. *bruguierana *ssp*. belangerana *against *Anopheles stephensi *larvae

**(%) Mortality**	**Total dead**	**Test sample** ^a^
20	28	**Total**
86	86	**Petroleum ether**
0	0	**Chloroform**
0	0	**Ethyl Acetate**
0	0	***n*** **-Butanol**
0	0	**Remaining methanol**
0	0	**Control (Methanol)**

^a^ : For each test sample, 4 replicates of 25 larvae were used

 According to the mortality data, only the total extract and petroleum ether fraction had larvicidal activity with mortality rate of 28% and 86%, respectively, at concentration of 40 ppm, while the other fractions were inactive. 

**Figure 1:**
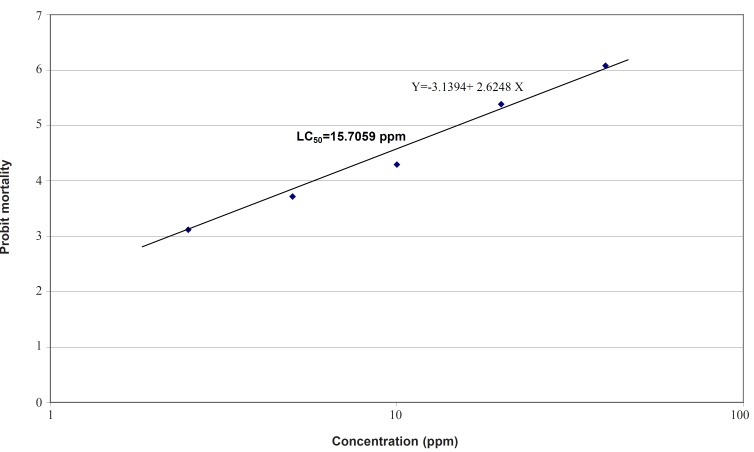
Probit regression line for response of *Anopheles stephensi *larvae to petroleum ether fraction treatment in laboratory tests.

Therefore, the logarithmic concentrations were subjected to larvicidal assay for these two test samples (Tables 2 and 3). The probit regression line for petroleum ether fraction is plotted in Figure 1 and LC_50_, LC_90_, confidence interval (CI) and slope values are presented in Table 4. For petroleum ether fraction, the LC_50_ (lethal concentration to cause 50% mortality in population) and LC_90 _(lethal concentration to cause 90% mortality in population) were measured as 15.70 ppm and 48.3 ppm, respectively (Table 4).

**Table 2:** Larvicidal activity of total methanolic extract of *C*. *bruguierana *ssp. *belangerana *against *Anopheles stephensi *larvae at logarithmic concentrations

**Mortality %**	**Total dead**	**Concentration ppm ** ^a^
28	28	40
12	12	20
4	4	10
0	0	5
0	0	2.5
0	0	Control (Methanol)

^a^ : For each concentration, 4 replicates of 25 larvae were used

**Table 3:** Larvicidal activity of petroleum ether fraction of *C*. *bruguierana *ssp. *belangerana *against *Anopheles stephensi *larvae at logarithmic concentrations

**Mortality (%)**	**Total dead**	**Concentration (ppm )** ^a^
86	86	40
65	65	20
24	24	10
10	10	5
3	3	2.5
0	0	Control (Methanol)

^a^: For each concentration, 4 replicates of 25 larvae were used

**Table 4:** Probit regression line parameters of the response of *Anopheles stephensi *larvae to petroleum ether fraction in laboratory tests

**Intercept**	**Slope ± SE**	**LC**_50_ ** (ppm)± 95% CI**	**LC**_90 _ **(ppm)± 95% CI**	**χ** ^2^	**χ** ^2^ **table (df)**	**p-value**
-3.1394	2.6248 ± 0.187	14.0035 15.7059 17.6504	40.3831 48.3433 60.6405	3.442*	7.81 (3)	< 0.05

*: No heterogeneity; SE: Standard Error; LC_50_: Lethal Concentration to cause 50% mortality in population; LC_90_: Lethal Concentration to cause 90% mortality in population; CI: Confidence Interval; χ^2^ (df) = heterogeneity about the regression line (Degrees Of Freedom)

On the basis of the presence of nonpolar compounds in petroleum ether fraction, we can assume that the larvicidal activity of this fraction would be related to these compounds. On the other hand, using the biopesticides containing active nonpolar compounds would not produce water pollution because of their accumulation on the outer surface of the water where the larvae spread in swamps.
